# RIG-I contributes to the innate immune response after cerebral ischemia

**DOI:** 10.1186/s12950-015-0101-4

**Published:** 2015-09-14

**Authors:** Frank J. Brand, Juan Carlos de Rivero Vaccari, Nancy H. Mejias, Ofelia F. Alonso, Juan Pablo de Rivero Vaccari

**Affiliations:** Department of Neurological Surgery, The Miami Project to Cure Paralysis, Miller School of Medicine, University of Miami, Miami, FL 33136 USA; Louisiana State University School of Medicine/Ochsner Medical Center – Ophthalmology Department, New Orleans, LA 70112 USA

**Keywords:** Innate immunity, Neuroinflammation, Stroke, RIG-I, ischemia

## Abstract

**Background:**

Focal cerebral ischemia induces an inflammatory response that when exacerbated contributes to deleterious outcomes. The molecular basis regarding the regulation of the innate immune response after focal cerebral ischemia remains poorly understood.

**Methods:**

In this study we examined the expression of retinoic acid-inducible gene (RIG)-like receptor-I (RIG-I) and its involvement in regulating inflammation after ischemia in the brain of rats subjected to middle cerebral artery occlusion (MCAO). In addition, we studied the regulation of RIG-I after oxygen glucose deprivation (OGD) in astrocytes in culture.

**Results:**

In this study we show that in the hippocampus of rats, RIG-I and IFN-α are elevated after MCAO. Consistent with these results was an increased in RIG-I and IFN-α after OGD in astrocytes in culture. These data are consistent with immunohistochemical analysis of hippocampal sections, indicating that in GFAP-positive cells there was an increase in RIG-I after MCAO. In addition, in this study we have identified n-propyl gallate as an inhibitor of IFN-α signaling in astrocytes.

**Conclusion:**

Our findings suggest a role for RIG-I in contributing to the innate immune response after focal cerebral ischemia.

## Background

Stroke is a major problem affecting populations worldwide. It is the second most common cause of death in the world after heart disease. In the United States stroke is the fourth leading cause of death, and the societal costs are approximately 80 billion dollars, which are expected to double by the year 2030 [1]. As a result, it is important to identify new and better therapies aimed at successfully treating this patient population.

Inflammation is a major contributor to the deleterious effects that present after an ischemic event such as stroke [2]. A component of inflammation is the innate immune response, which is characterized by the activation of pattern recognition receptors (PRR) such as Toll-like receptors (TLRs), NOD-like receptors (NLRs) or RIG-like receptors (RLRs). TLRs have been previously studied in regards to their deleterious and beneficial effects following stroke [3–8]. Similarly NOD-like receptors (NLRs), such as NLRP1 or NLRP3, have been shown to contribute to the inflammatory response after stroke through the formation of inflammasomes [9–13]. However, not much is known about the contribution of RLR signaling to the pathology after focal cerebral ischemia.

RLR signaling is typically involved in the production of type I IFNs such as IFN-α following RNA viral infections. We have previously shown that in the central nervous system (CNS), RLR signaling contributes to the process of reactive astrogliosis following spinal cord injury [14], and that retinoic acid-inducible gene-I (RIG-I), an RLR PRR, is elevated in the cortex of patients with early signs of Alzheimer’s disease (mild cognitive impairment), contributing to the production of amyloid precursor protein and amyloid-β [15]. However, the role of RIG-I to the regulation of inflammation after cerebral ischemia remains unexplored.

In the present project, we have studied the involvement of RIG-I after focal cerebral ischemia in rats. Here we also show the effects of oxygen glucose deprivation (OGD) on RIG-I signaling activation in astrocytes and identify n-propyl gallate (nPG) as an inhibitor of IFN-α in astrocytes.

## Materials and methods

### Animals and focal cerebral ischemia

All animal procedures were approved by the Institutional Animal Care and Use Committee of the University of Miami (protocol number 12–200). To induce transient middle cerebral artery occlusion (MCAO), male Sprague–Dawley rats were anesthetized with 3 % isoflurane in a mixture of 70 % N_2_O/30 % O_2_ for 5 min. Rats were intubated and immobilized with pancuronium bromide (0.35 mg/kg, IV) and mechanically ventilated. Brain and body temperature were monitored and the animals were maintained at a normothermic (37 °C) temperature. A catheter was implanted into the tail artery for blood-gas monitoring during surgery. The right common carotid artery was exposed with a midline neck incision and dissected from the surrounding nerves. The external carotid artery and superior thyroid artery were then ligated. A 4-cm length of 3–0 suture that was heat-blunted with a 20 mm section coated with poly-l-lysine solution (0.1 % wt/vol) was used for the occlusion. This suture was inserted at the proximal external carotid artery into the internal carotid artery to the MCA. The MCA was occluded for 90 min, then released for reperfusion. The neck incision was closed with staples and the animals were allowed to wake-up from the anesthesia. The animal was then taken to a cage supplied with food and water until termination of the study. Animals that presented with difficulty eating, presented blood pressure abnormalities, respiratory difficulties or brains that were not well perfused were euthanized and excluded from the study.

### Immunoblotting

For detection of RLR proteins after MCAO, protein lysates ipsilateral to the ischemic side, of hippocampal brain areas were obtained and resolved by immunoblotting. We did not detect any effects of MCAO on RIG-I signaling on the contralateral side (data not shown). Sham animals were used as controls. Animals were sacrificed at different time points after ischemia (1 h, 4 h, 1d and 3d after MCAO) and then lysates were prepared and resolved by immunoblotting as described in de Rivero Vaccari *et al*. [16] using antibodies against RIG-I (1:1000, Anaspec), IFN-α (1:1000, Abcam) and β-actin (1:5000, Sigma). Data was normalized to β-actin.

### Perfusion fixation

For immunohistochemical analysis rats were anesthetized and perfusion-fixed with 4 % paraformaldehyde (PFA) as described in de Rivero Vaccari et al. [16].

### Immunohistochemistry and confocal microscopy

Immunostained brain sections of sham and 4 h MCAO animals were examined with an Olympus FV-1000 Laser Scanning Confocal Microscope (Olympus). Rats were perfused with 4 % paraformaldehyde as described [16] and processed for cryostat sectioning (Leica SM 2000R Sliding Microtome). Sections (50 μm) were prepared for immunohistochemical analysis as described in de Rivero Vaccari et al. [16] with a primary antibody to RIG-I (1:500, Abcam) and with the astrocytic marker anti-glial fibrillary acidid protein (GFAP) (Millipore Bioscience Research Reagents). Alexa-Fluor secondary antibody conjugates (1:500, Invitrogen) were used. Secondary antibodies alone were used as control for antibody specificity.

### Astrocyte culture preparation

Primary rat astrocyte cells (Lonza) were plated and grown in culture for 7 days prior to experimentation at a density of 4×10^4^ cells/9.5 cm^2^ as described in de Rivero Vaccari *et al.* [14]. Cells were grown in Astrocyte Basic Medium (Lonza).

### Oxygen Glucose Deprivation (OGD)

Astrocyte cultures were grown as described above. For OGD, cultures were exposed to 95 % nitrogen and 5 % CO_2_ atmosphere maintained by constant gas flow at 37 °C in all experiments. Oxygen tension was kept at 5 % during the duration of the experiment. The Nitrogen/CO2 content was controlled with the ProOx P110 Oxygen Controller with an E702 Oxygen Sensor (model #P-110-E70) with single set point controller (#P110) (Biospherix). The media used in these OGD experiments consisted of hypoglycemic medium containing normal salts, 1 mg/mL bovine serum albumin (BSA) and 55 μM glucose (1 % of the normal glucose concentration). Control groups were grown under normal culture conditions (5 % CO_2_ and 95 % Oxygen).

### NF-κB activation assay

To test for NF-κB activation, protein lysates (Control, 2, 3 and 4 h after OGD) were obtained from rat astrocytes and assayed with the PathScan Phospho-NF-κB p65 (Ser536) Sandwich Elisa kit (Cell Signaling) according to manufacturer’s instructions. Briefly, 100 μl of cell lysate were added and sealed to the appropriate wells for a 2 h incubation period at 37 °C. Each well was washed 4 times with 1X Wash Buffer. A detection antibody was added to each well and incubated at 37 °C for 1 h. Then 100 μl of reconstituted HRP-Linked secondary antibody were added to each well and incubated for 30 min followed by adding 100 μl of TMB Substrate to each well for 10 min and a STOP solution. The plate was read using a spectrophotometer (Victor^3^ 1420 multilabel counter, Perkin Elmer) at 450 nm.

### Cell reporter assay for RLR signaling activation in astrocytes

Astrocytes were grown in culture as described above in a 96-well plate. RLR signaling was stimulated in cells with poly(I:C)LMW (low molecular weight) at 3 different concentrations (1, 3 and 6 μg/ml, dissolved in sterile water) for 24 h. On day 2, 150 μl per well of B16-BLUE IFN-α/β cells (Invivogen) in suspension (50,000 cells) were added and incubated overnight at 37 °C in 5 % CO_2_. On day 3, 50 μl per well of the supernatant were added to 150 μl of QUANTI-Blue (Invivogen). The plate was incubated for 2 h at 37 °C and read at 600 nm in a spectrophotometer (Victor^3^ 1420 multilabel counter, Perkin Elmer). A similar procedure was carried to test n-propyl-gallate (n-PG, dissolved in dimethyl sulfoxide (DMSO), MP Biomedicals) as a blocker of type I IFN signaling at different concentrations (5, 10 and 50 μM).

### Statistical analysis

Statistical comparisons between the different groups were done using a two-tailed Student’s *t*-test or a one-way ANOVA followed by Dunnett’s multiple comparison tests. P-values of significance used were *P* < 0.05.

## Results

### RLR signaling protein expression is altered in the hippocampus of rats after MCAO

**Fig. 1:**
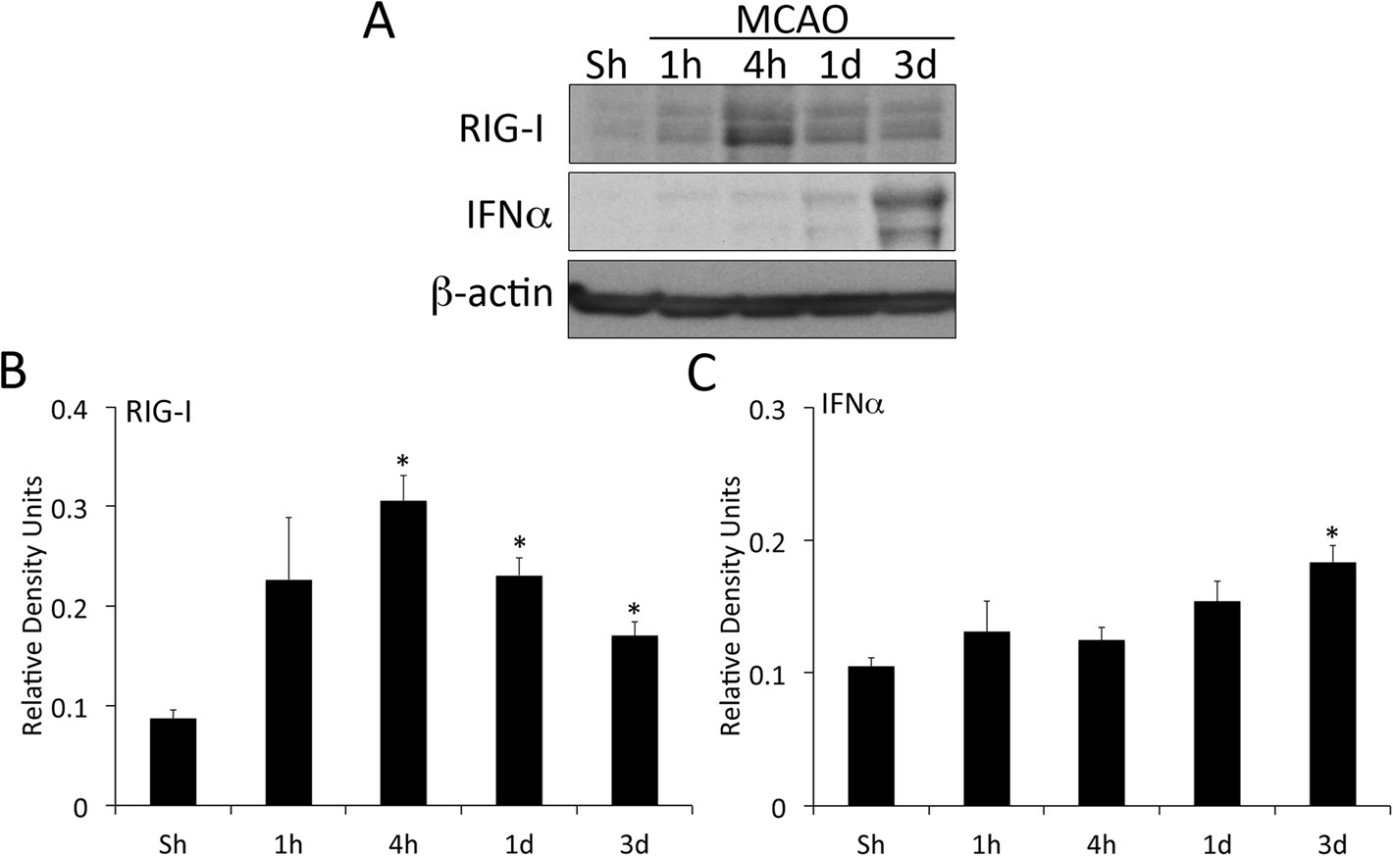
MCAO alters RLR signaling protein expression in the hippocampus of rats. Representative immunoblot analysis of hippocampal lysates of sham (Sh) and rats sacrificed at 1 h, 4 h, 1d and 3d after MCAO (**a**). Hippocampal lysates corresponding to the ipsilateral side of the brain to MCAO were immunoblotted with antibodies against (**b**) RIG-I and (**c**) IFN-α. Data presented as mean+/−SEM. *p < 0.05. *N* = 5 per group

To determine if RLR protein expression is altered after MCAO, we analyzed protein lysates obtained from the hippocampus (ipsilateral side to MCAO) of rats at different time points after ischemia (1 h, 4 h, 1d and 3d) and in sham-operated animals for the expression of RIG-I and IFN-α (Fig. 1). MCAO resulted in increased protein expression of RIG-I at 4 h, 1d and 3d (Fig. 1b) as well as of IFN-α at 3d (Fig. 1c) in the hippocampus of rats. These data indicate an involvement of RIG-I after focal cerebral ischemia.

### MCAO induces alterations in RIG-I protein expression in astrocytes

**Fig. 2:**
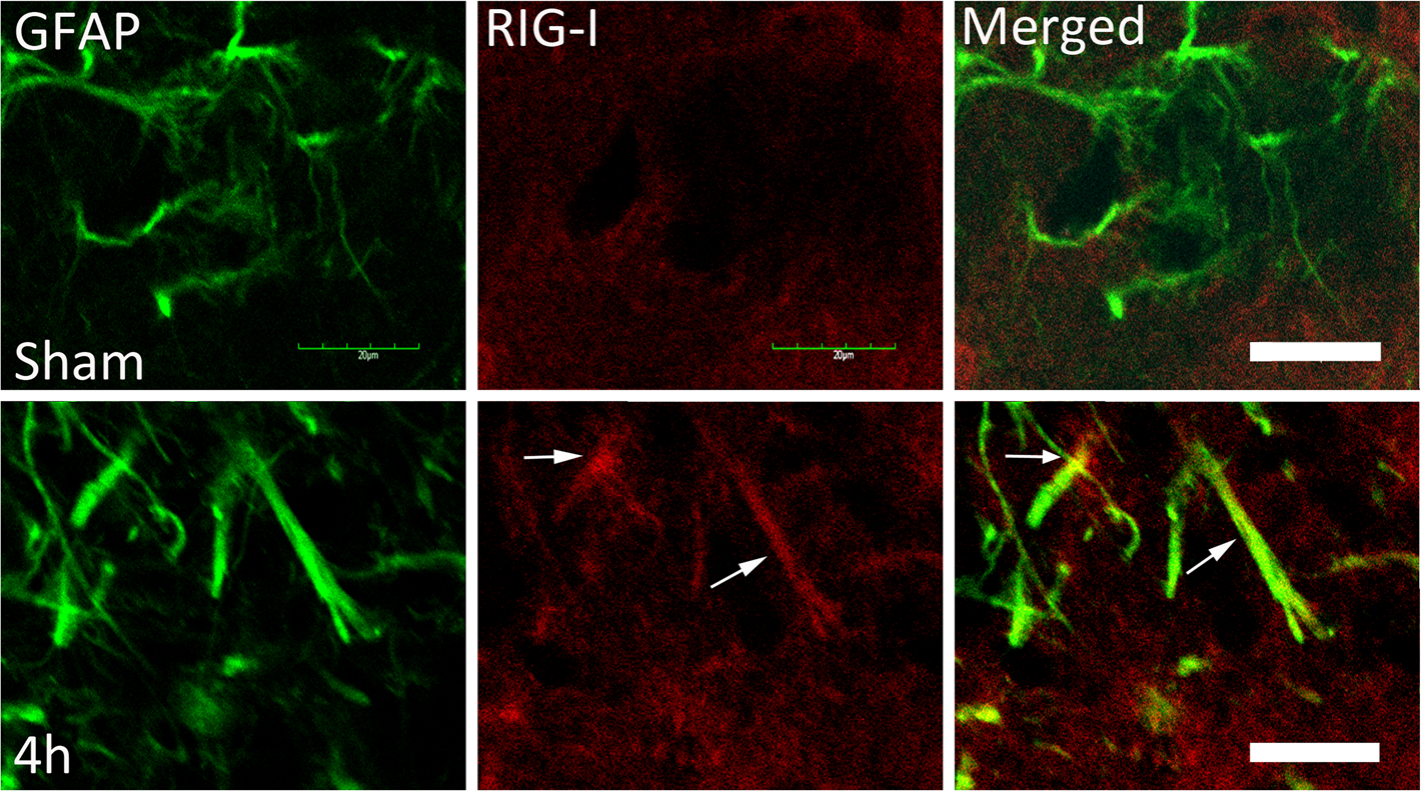
MCAO increases RIG-I immunoreactivity in astrocytes. Confocal images corresponding to the hippocampus of sham animals (top) and animals that were sacrificed 4 h after MCAO (bottom). Image is taken from the side of the brain ipsilateral to MCAO. Sections were stained for RIG-I (red) and GFAP (green). Arrows point to RIG-I positive astrocytes. Scale bar = 20 μm

Confocal images of frozen sections were double stained with RIG-I (A, red) and the astrocyte marker GFAP (green). Four hours after MCAO, there was an increased in the immunoreactivity of RIG-I in astrocytes (arrows) when compared to sham-operated animals (Fig. 2). These findings indicate that RIG-I expression increased after MCAO in astrocytes after ischemia.

### OGD induces RLR signaling components in astrocytes

**Fig. 3:**
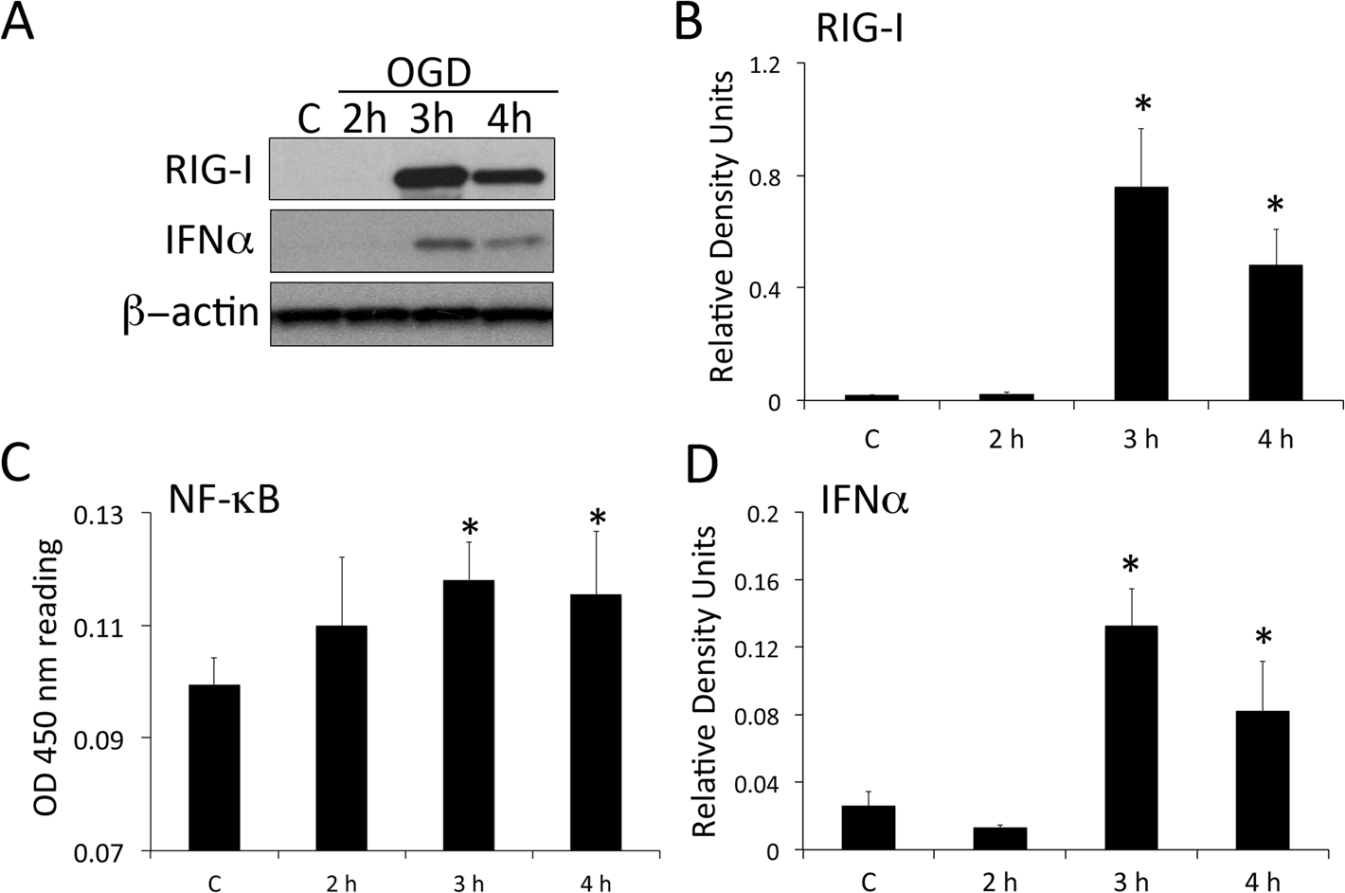
OGD activates RLR signaling in primary astrocytes in culture. Representative immunoblot analysis of astrocyte lysates of control (**c**) and astrocytes subjected to OGD and then harvested at 2 h, 3 h and 4 h (**a**). Cell lysates were immunoblotted with antibodies against (**b**) RIG-I and (**c**) IFN-α. β-Actin was used as a standard and control for protein loading. **d** Bar graph showing NF-κB activity following OGD in astrocytes. Data presented as mean+/−SEM. *p < 0.05. *N* = 6 per group

Since RIG-I protein expression is increased by ischemia in astrocytes (Fig. 2), we wanted to establish whether OGD induces RLR signaling in astrocytes in culture. Primary astrocytes were grown in culture and subjected to OGD for 2, 3 and 4 h. Cells grown under standard culture conditions (no OGD) were used as a control (C). As shown in Fig. 3, there is a statistically significant increase in RIG-I and IFN-α at 3 and 4 h after OGD in astrocytes in culture. Consistent with these results is the activation of NF-κB at 3 and 4 h as determined by an NF-κB activation assay. NF-κB is involved in the production of type I IFNs such as IFNα. This is consistent with well-established evidence indicating that NF-κB is activated after cerebral ischemia [17, 18]. The levels of phosphorylated IRF3/7 did not differ among the different groups tested (data not shown). These results indicate that RIG-I signaling is increased in astrocytes by OGD, consistent with increased expression of NF-κB and IFN-α.

### Propyl Gallate (n-PG) inhibits RLR signaling in primary astrocytes

**Fig. 4:**
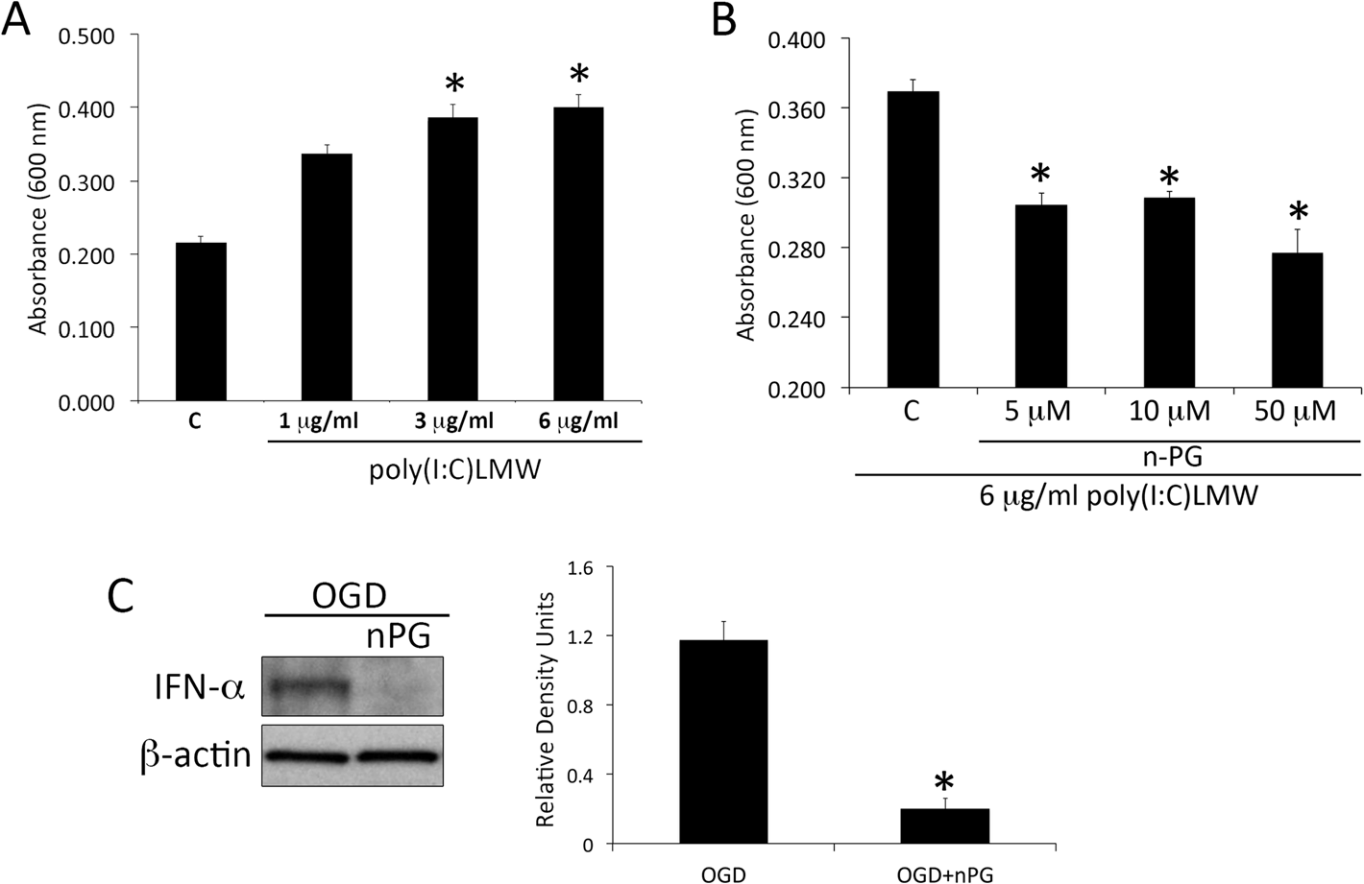
n-PG inhibits RLR signaling in primary astrocytes. **a** Bar-graph showing the results of a cell reporter assay for the activation of RLR signaling activation/production of IFN-α/β following stimulation of primary rat astrocytes with the RLR ligand poly(I:C)LMW at a concentration of 1, 3 or 6 μg/ml for 18 h. C = control (untreated cells). **b** Bar-graph showing the results of a cell reporter assay for the activation of RLR signaling activation/production of IFN-α/β following stimulation of primary rat astrocytes with the RLR ligand poly(I:C)LMW at a concentration of 6 μg/ml and treated with n-PG at a concentration of 5, 10, 50 μM for 18 h. C = control (untreated cells). **c** Representative immunoblot analysis of astrocyte lysates of cells in culture subjected to OGD (4 h) and astrocytes pretreated with 50 μM n-PG for 2 h and then subjected to OGD. Cell lysates were immunoblotted with an antibody against IFN-α. β-Actin was used as a standard and control for protein loading. Data presented as mean+/−SEM. *p < 0.05. *N* = 6 OGD and 8 OGD + nPG

To identify a potential inhibitor of RLR signaling, we used a cell reporter assay that produces IFN-α/β after stimulation. We stimulated astrocytes in culture with the RLR ligand poly(I:C)LMW for 18 h at concentrations of 1, 3 and 6 μg/ml. Our findings indicate that at concentrations of 3 and 6 μg/ml of poly(I:C)LMW, RLR signaling increased (Fig. 4a). Then we stimulated cells in a similar manner with 6 μg/ml of poly(I:C)LMW combined with n-PG treatment at concentrations of 5, 10 and 50 μM. At all of these concentrations n-PG was able to lower RLR signaling activation when compared to the poly(I:C)LMW-treated/n-PG-untreated group, as determined by the cell reporter assay (Fig. 4b). In addition, astrocytes were pretreated with 50 μM n-PG for 2 h prior to 4 h of OGD. The 50 μM, 2 h n-PG-treatment resulted in decreased IFN-α production (Fig. 4c); thus suggesting that n-PG can be used to inhibit RLR signaling in astrocytes after ischemia.

## Discussion

Worldwide, stroke is the second most common cause of death after heart disease. Despite its prevalence, the therapies available for stroke patients remain limited and in most instances do not offer significant improvements. As a result, it is important to identify new targets that can be used for the development of treatments for cerebral ischemia. A promising target for the treatment of stroke is the inflammatory innate immune response.

The involvement of PRR such as TLR and NLR in brain ischemia has been previously described [11, 13, 19–23]. In the present study we extend the knowledge regarding the role of PRR in brain ischemia by studying the effects of focal cerebral ischemia on RIG-I expression after MCAO. RIG-I is a RLR involved in the process of reactive astrogliosis after spinal cord injury [14]. In this study we have identified RIG-I as a PRR altered by focal cerebral ischemia in the hippocampus of rats after MCAO. When looking at whole hippocampal proteins lysates, RIG-I protein expression increased after MCAO. The increase in RIG-I expression was consistent with the increase in IFN-α. When analyzing the hippocampus of rats in immunohistochemical sections after MCAO, we detected increased immunoreactivity of RIG-I in astrocytes, suggesting that indeed RIG-I is increased after MCAO in the hippocampus. Moreover, our data in culture indicate that in astrocytes, RIG-I expression increased after OGD, which is consistent with our immunohistochemistry findings in GFAP-positive cells *in vivo*. This increase in RIG-I by OGD in cultured astrocytes was consistent with an increase in IFN-α expression, which is produced in a NF-κB dependent manner. In addition, *in vivo,* RIG-I expression was more prevalent in astrocytes.

Propyl gallate has been previously shown to be beneficial in the treatment of dilated cardiomyopathy in mice [24], and it is usually found as a food additive to prevent oxidation due to its strong anti-oxidant effects [25]. At the same time, n-PG has anti-inflammatory effects by down-regulating NF-κB or by suppressing the phosphorylation of c-Jun NH(2)-terminal kinase 1/2 (JNK1/2) [26, 27]. Consistent with these findings, in the present study, we have identified n-PG as an inhibitor of IFN-α signaling in astrocytes. Future studies should also look into more specific blockers of RIG-I or should use RIG-I knockout animals to better elucidate the contribution of RIG-I to the pathology of brain ischemia.

## Conclusion

To the best of our knowledge, this is the first report to indicate an involvement of RIG-I in the inflammatory response after stroke, and we have identified n-PG as a potential anti-inflammatory drug after cerebral ischemia. Whether RLR signaling is beneficial or detrimental after stroke is under investigation. However, it has been suggested that inhibition of type I IFN signaling is beneficial in reducing hypoxia-induced neuroinflammation [28]. In contrast, other reports indicate that delivery of IFN-β has anti-inflammatory properties after ischemic stroke in rats by decreasing infiltration of neutrophils and monocytes into the brain [29]. As a result, the role of RLRs and type I IFN signaling after cerebral ischemia needs further investigation in order to obtain a better idea regarding the potential therapeutic potential of targeting RIG-I signaling under these conditions.

## References

[1] Corbyn Z (2014). Statistics: a growing global burden. Nature.

[2] Danton GH, Dietrich WD (2003). Inflammatory mechanisms after ischemia and stroke. J Neuropathol Exp Neurol.

[3] Brea D, Blanco M, Ramos-Cabrer P, Moldes O, Arias S, Perez-Mato M (2011). Toll-like receptors 2 and 4 in ischemic stroke: outcome and therapeutic values. J Cereb Blood Flow Metab.

[4] Brea D, Blanco M, Sobrino T, Ramos-Cabrer P, Castillo J (2011). The levels of expression of toll-like receptors 2 and 4 in neutrophils are associated with the prognosis of ischaemic stroke patients. Rev Neurol.

[5] Brea D, Sobrino T, Rodriguez-Yanez M, Ramos-Cabrer P, Agulla J, Rodriguez-Gonzalez R (2011). Toll-like receptors 7 and 8 expression is associated with poor outcome and greater inflammatory response in acute ischemic stroke. Clin Immunol.

[6] Downes CE, Crack PJ (2010). Neural injury following stroke: are Toll-like receptors the link between the immune system and the CNS?. Br J Pharmacol.

[7] Fadakar K, Dadkhahfar S, Esmaeili A, Rezaei N (2014). The role of Toll-like receptors (TLRs) in stroke. Rev Neurosci.

[8] Leung PY, Packard AE, Stenzel-Poore MP (2009). It's all in the family: multiple Toll-like receptors offer promise as novel therapeutic targets for stroke neuroprotection. Future Neurol.

[9] Fann DY, Santro T, Manzanero S, Widiapradja A, Cheng YL, Lee SY (2014). Intermittent fasting attenuates inflammasome activity in ischemic stroke. Exp Neurol.

[10] de Rivero Vaccari JP, Dietrich WD, Keane RW (2014). Activation and regulation of cellular inflammasomes: gaps in our knowledge for central nervous system injury. J Cereb Blood Flow Metab.

[11] Fann DY, Lee SY, Manzanero S, Tang SC, Gelderblom M, Chunduri P (2013). Intravenous immunoglobulin suppresses NLRP1 and NLRP3 inflammasome-mediated neuronal death in ischemic stroke. Cell Death Dis.

[12] Fann DY, Lee SY, Manzanero S, Chunduri P, Sobey CG, Arumugam TV (2013). Pathogenesis of acute stroke and the role of inflammasomes. Ageing Res Rev.

[13] Abulafia DP, de Rivero Vaccari JP, Lozano JD, Lotocki G, Keane RW, Dietrich WD (2009). Inhibition of the inflammasome complex reduces the inflammatory response after thromboembolic stroke in mice. J Cereb Blood Flow Metab.

[14] de Rivero Vaccari JP, Minkiewicz J, Wang X, De Rivero Vaccari JC, German R, Marcillo AE (2012). Astrogliosis involves activation of retinoic acid-inducible gene-like signaling in the innate immune response after spinal cord injury. Glia.

[15] de Rivero Vaccari JP, Brand FJ, Sedaghat C, Mash DC, Dietrich WD, Keane RW (2014). RIG-1 receptor expression in the pathology of Alzheimer's disease. J Neuroinflammation.

[16] de Rivero Vaccari JP, Lotocki G, Alonso OF, Bramlett HM, Dietrich WD, Keane RW (2009). Therapeutic neutralization of the NLRP1 inflammasome reduces the innate immune response and improves histopathology after traumatic brain injury. J Cereb Blood Flow Metab.

[17] Clemens JA, Stephenson DT, Smalstig EB, Dixon EP, Little SP (1997). Global ischemia activates nuclear factor-kappa B in forebrain neurons of rats. Stroke.

[18] Terai K, Matsuo A, McGeer EG, McGeer PL (1996). Enhancement of immunoreactivity for NF-kappa B in human cerebral infarctions. Brain Res.

[19] Hua F, Ma J, Ha T, Kelley JL, Kao RL, Schweitzer JB (2009). Differential roles of TLR2 and TLR4 in acute focal cerebral ischemia/reperfusion injury in mice. Brain Res.

[20] Bohacek I, Cordeau P, Lalancette-Hebert M, Gorup D, Weng YC, Gajovic S (2012). Toll-like receptor 2 deficiency leads to delayed exacerbation of ischemic injury. J Neuroinflammation.

[21] Cao CX, Yang QW, Lv FL, Cui J, Fu HB, Wang JZ (2007). Reduced cerebral ischemia-reperfusion injury in Toll-like receptor 4 deficient mice. Biochem Biophys Res Commun.

[22] Caso JR, Pradillo JM, Hurtado O, Lorenzo P, Moro MA, Lizasoain I (2007). Toll-like receptor 4 is involved in brain damage and inflammation after experimental stroke. Circulation.

[23] Pradillo JM, Fernandez-Lopez D, Garcia-Yebenes I, Sobrado M, Hurtado O, Moro MA (2009). Toll-like receptor 4 is involved in neuroprotection afforded by ischemic preconditioning. J Neurochem.

[24] Du CK, Zhan DY, Morimoto S (2014). In vivo effects of propyl gallate, a novel Ca(2+) sensitizer, in a mouse model of dilated cardiomyopathy caused by cardiac troponin T mutation. Life Sci.

[25] Final report on the amended safety assessment of Propyl Gallate. Int J Toxicol. 2007;26 Suppl 3:89–118. doi:10.1080/10915810701663176. 10.1080/10915810701663176 18080874

[26] Hsu HC, Lin WC, Chang PJ, Hong CZ, Chen CH (2013). Propyl gallate inhibits TPA-induced inflammation via the nuclear factor-kappaB pathway in human THP-1 monocytes. Exp Ther Med.

[27] Jung HJ, Kim SJ, Jeon WK, Kim BC, Ahn K, Kim K (2011). Anti-inflammatory activity of n-propyl gallate through down-regulation of NF-kappaB and JNK pathways. Inflammation.

[28] Minter MR, Zhang M, Ates RC, Taylor JM, Crack PJ (2014). Type-1 interferons contribute to oxygen glucose deprivation induced neuro-inflammation in BE(2)M17 human neuroblastoma cells. J Neuroinflammation.

[29] Veldhuis WB, Derksen JW, Floris S, Van Der Meide PH, De Vries HE, Schepers J (2003). Interferon-beta blocks infiltration of inflammatory cells and reduces infarct volume after ischemic stroke in the rat. J Cereb Blood Flow Metab.

